# Supplementary material for: Nuclear HKII-P-p53 (Ser15) Interaction is a Prognostic Biomarker for Chemoresponsiveness and Glycolytic Regulation in Epithelial Ovarian Cancer
Source: Cancers (Basel). 2021 Jul 7;13(14):3399. doi: 10.3390/cancers13143399 (PMC8306240; doi:10.3390/cancers13143399)
Supplement: Supplementary file 1 [file cancers-13-03399-s001.zip › cancers-1276270-supplementary/suppl/cancers-1276270-supplementary-.pdf]

Supplementary Materials: Nuclear HKII–P-p53 (Ser15) Interaction is a Prognostic Biomarker for Chemoresponsiveness and Glycolytic Regulation in Epithelial Ovarian Cancer

Chae Young Han, David A Patten, Se Ik Kim, Jung Jin Lim, David W Chan, Michelle KY Siu, Youngjin Han, Euridice Carmona, Robin J. Parks, Cheol Lee, Li-Jun Di, Zhen Lu, Karen K. L. Chan, Ja-Lok Ku, Elizabeth A. Macdonald, Barbara C. Vanderhyden, Anne-Marie Mes-Masson, Hextan Y.S. Ngan, Annie N. Y. Cheung, Yong Sang Song, Robert C. Bast Jr., Mary-Ellen Harper and Benjamin K. Tsang

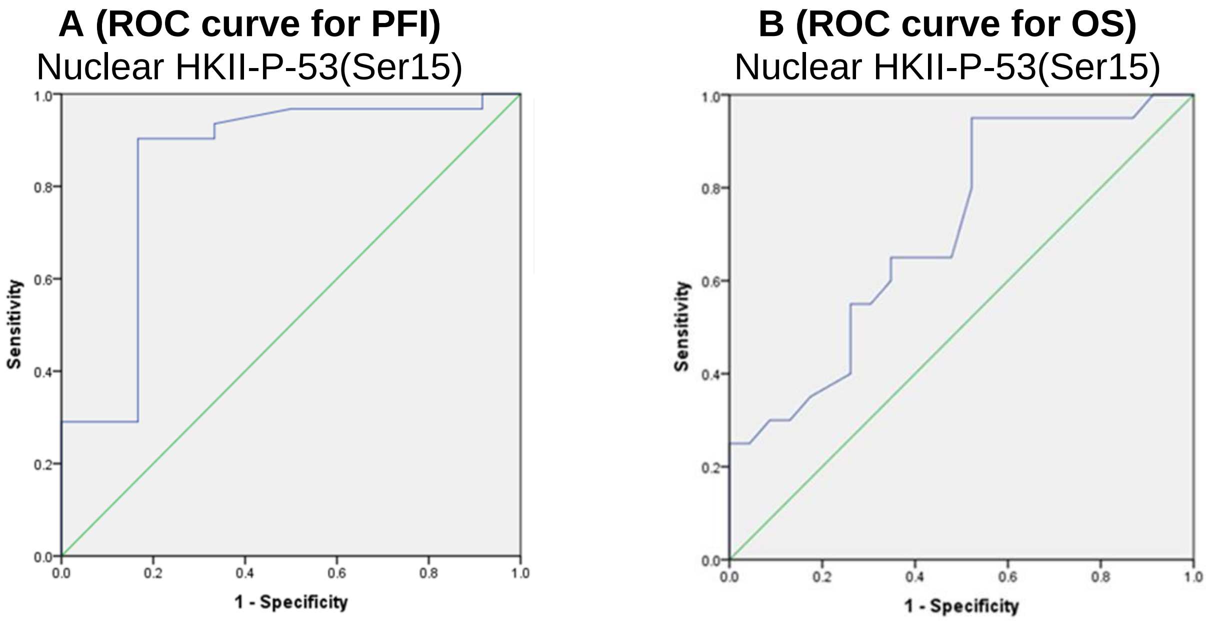

Parameter	Progression Free Interval (PFI)	Overall Survival (OS)
Area Under the Curve (AUC)	0.844	0.708
Significance (*p*)	0.001	0.020
95% Confidence Interval (CI)	0.690-0.999	0.552-0.863
n	n = 41	n =40

Figure S1. Receiver Operating Characteristic (ROC) curves for (A) Progression Free Interval (PFI) and (B) Overall survival (OS). ROC curves for (A) Progression Free Interval (PFI) and (B) Overall survival (OS) were computed with SPSS to determine nuclear HKII-P-p53(Ser15) as potential biomarker for predicting chemoresponsiveness as PFI and OS. Area Under the Curve (AUC) for PFI is 0.844 (95% CI, 0.690-0.990, $p = 0.001$) and AUC for OS is 0.708 (95% CI, 0.552-0.863, $p = 0.020$). *p* refers significance. CI refers Confidence Interval.

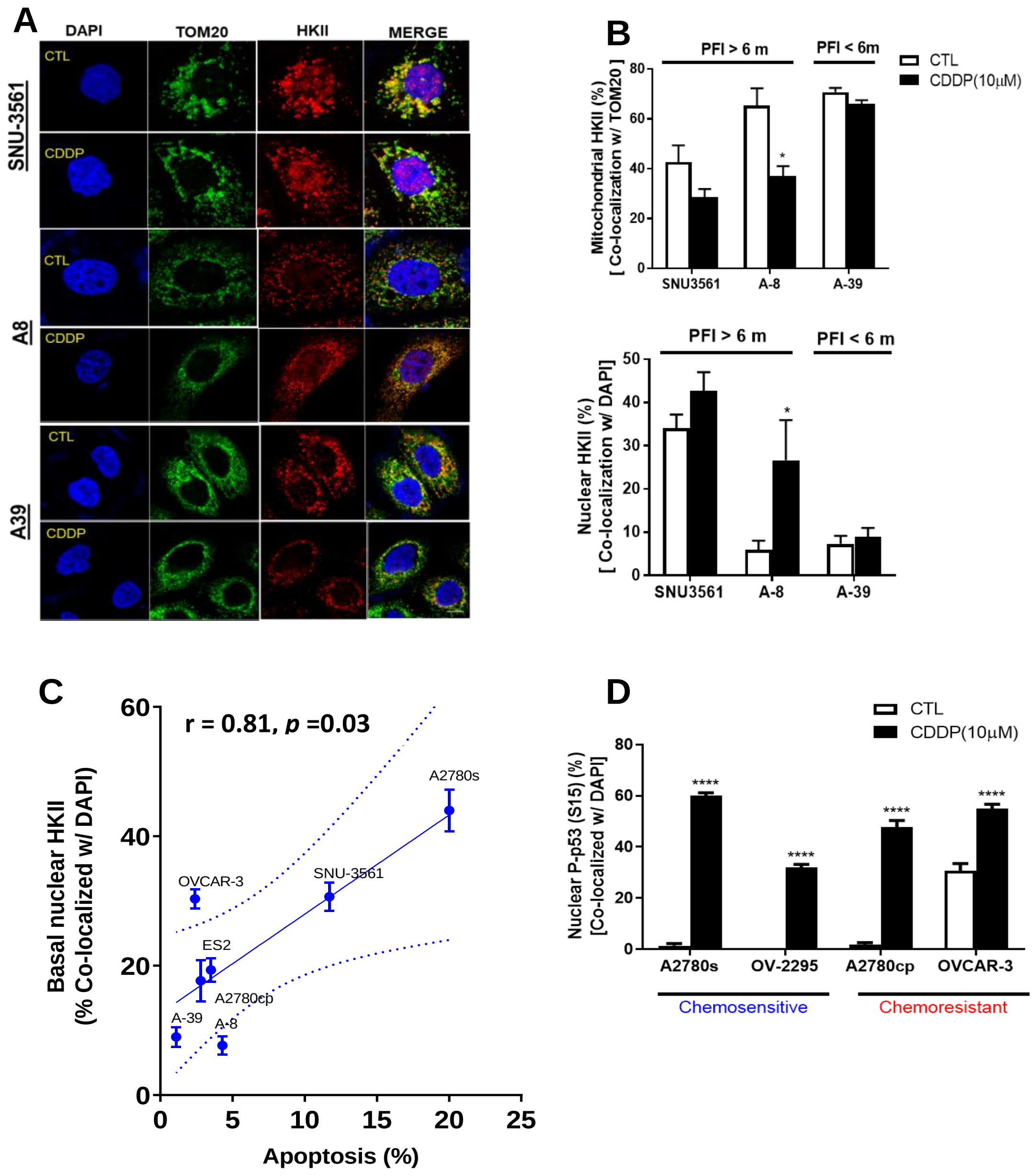

Figure S2. Nuclear HKII localization in primary EOC cultures. **(A)** Primary ovarian cultures from epithelial ovarian cancer (EOC) patients with different sub-types, high grade serous (HGS) SNU-3561 and ascites (A-8, HGS), and primary EOC cells (A-39, clear cell, CC) were cultured with CDDP (10μM, 24 h). Cellular localization of HKII (Red) as % of total cells with TOM20 (Green: mitochondrial Marker) and DAPI (Blue: Nucleus marker) were measured using confocal microscopy **(B)** Results were quantified using Image premier program and progression free interval (PFI) is shown in each cell. **(C)** Correlation between the basal nuclear HKII level (CDDP untreated) in cell lines and primary EOC cells/ascites and CDDP-induced apoptosis was measured. Apoptosis was assessed by Hoechest nuclear staining. **(D)** Nuclear P-p53 (S15) localization in chemosensitive and chemoresistant cells cultured with and without CDDP (10μM, 24 h) was measured. Error bars denote ± SEM (n = 5). (* $p < 0.05$, CTL vs. CDDP). Scale bar: 10 μm.

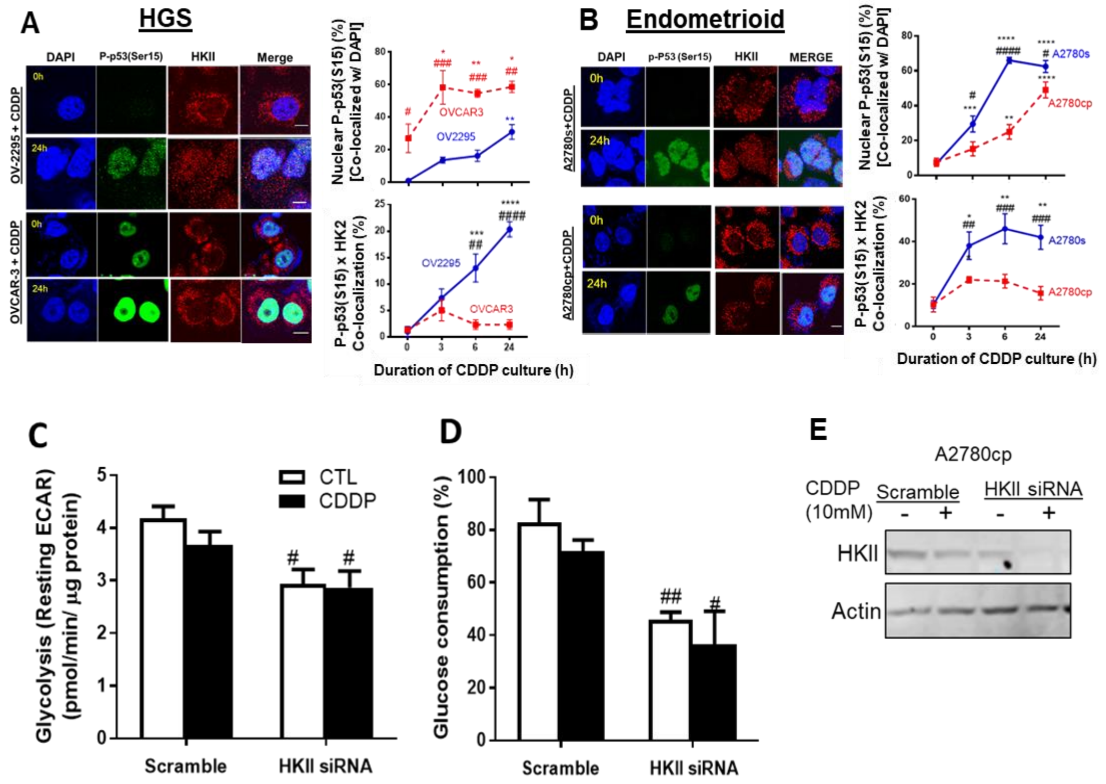

Figure S3: Mito-HKII is associated with chemoresistance and metabolism in EOC. **(A)** **(B)** EOC cell lines (OV-2995, OVCAR-3, A2780s, and A2780cp) were cultured with CDDP (0,10μM, 6-24h) and examined with confocal microscopy. Cellular localization of HKII (Red; as % of total cells), P-p53 Ser15 (Green), and DAPI (Blue: Nucleus marker) were analyzed with an Image Premier program. **(C)** Resting ECAR was measured with A2780cp cells transfected with 0, 100 nM of HKII siRNA and treated with CDDP (0, 10 μM, 24 h) using an XF96e Extracellular Flux Analyzer. **(D)** Glucose consumption was also measured using the same cells seeded on to 12-well plate followed by CDDP treatment (10 μM, 24 h). **(E)** HKII knocked down with siRNA was confirmed by WB. Error bars denote ± SEM (n = 4). (**p* < 0.05, ***p* < 0.01, ****p* < 0.001, *****p* < 0.0001, CTL vs. CDDP; # *p* < 0.05, ## *p* < 0.01 ### *p* < 0.001, #### *p* < 0.0001, A2780s vs. A2780cp).

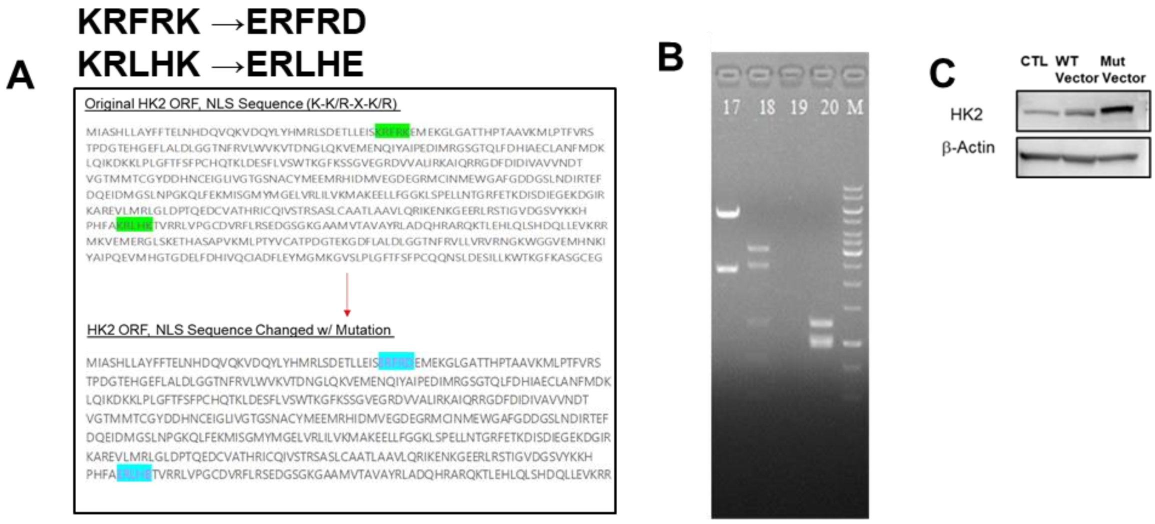

Figure S4. NLS site of HKII is critical for nuclear localization and apoptotic reaction. **(A)** Nuclear localization site (NLS) sites of HKII were mutated (KRFRK to EFRFD, KRLHK to ERLHE) as indicated and its mutation was confirmed as **(B)** showing different digestion pattern by restriction enzyme digestion (KpnI+ClaI) in A2780s cells. A2780s cells were transfected with CMV vector control, HKII WT and HKII NLS mutant vector (2.0μg, 24h). Transfection efficiency was confirmed as shown in **(C)** for Western blot.

Table S1. Information of EOC cell lines. EOC used in present studies are described depending on p53 status, other mutation sites, and chemosensitivity based on previous sequencing data and experiments for apoptotic rate measurement (Abedini et al., 2014; Fraser et al., 2008). Characterization of these cell lines are also verified in previous literature (Anglesio et al.,2013; Leroy et al., 2014; Domcke et al 2013).

Cell line	Tumor origin (Sub-type)	*TP53* status	Other mutation	Chemosensitivity
**A2780s**	endometrioid adenocarcinoma	Wild type	*PTEN/ARID1A*	Sensitive
**A2780cp**	endometrioid adenocarcinoma	Mutant<br>V127F (Exon5)<br>R260S (Exon8)	*PTEN/ARID1A*	Resistant
**OV-2295**	High grade serous (HGS)	Mutant<br>p.Ile195Thr (c.584T>C)	*PI3KCIA/ARID1A*	Sensitive
**OVCAR-3**	HGS	Mutant<br>R248Q	None Detected	Resistant
**ES-2**	clear cell carcinoma	Mutant<br>S241F	*BRAF*	Resistant

Table S2. Demographic patient information for primary human EOC cell/ascites cultures. The clinical characteristics of the patients recruited for primary ovarian cancer cultures of Ottawa Hospital Research Institute (OHRI) and Seoul National University (SNU). The majority of collected tumors were primarily HGS with one CC sub-type. Most of the tumors were at stage III and IV, although some received neo-adjuvant treatment that precluded accurate staging. For platinum sensitivity, the majority of patient had disease progression or recurrence within 6 months after treatment (progression free interval, PFI ≤ 6 months, m) as resistant patient, while four patients had a PFI of longer than 6 months (PFI > 6m, sensitive). Some of primary human cells (Untreated) were not previously exposed to chemotherapy before its collection whereas treated refers cells collected from patient after chemotherapy.

No.	Source	Histologic sub-type	Stage	Platinum sensitivity	PFI (month)	Survival status	Chemo status	Inst.
**SNU-3561**	Pleural fluid	HGS	4b	Sensitive	19	Alive	Untreated	SNU
**A-8**	Ascites	HGS	3c	Sensitive	39	Alive	Untreated	
**A-39**	Ascites	CC	Unknown	Resistant	2	Deceased	Treated	
**2068**	Ascites	HGS	3c	Resistant	0	Deceased	Treated	OHRI
**2149**	Ascites	HGS	1c	Sensitive	25	Alive	Treated	
**2159**	Ascites	HGS	Neo adjuvant (Unknown)	Resistant	0	Deceased	Treated	
**2161**	Ascites	HGS	Neo adjuvant (Unknown)	Sensitive	10	Unknown	Treated	
**2164**	Ascites	HGS	Neo adjuvant (Unknown)	Resistant	0	Deceased	Treated	
**2165**	Ascites	HGS	3c	Resistant	5	Alive	Treated	
**2166**	Ascites	HGS	Neo adjuvant (Unknown)	Resistant	4	Deceased	Treated	
**2170**	Ascites	HGS	3b	Resistant	4	Deceased	Treated	

Table S3. Demographic Information of EOC patients for immunohistochemistry. The clinical characteristics of the patients recruited of ovarian tumor formalin-fixed-paraffin-embedded (FFPE) sections used in proximity ligation assay (PLA) studies. Pre-chemotherapy and post-chemotherapy ovarian tumor sections were obtained at primary and secondary cytoreductive surgery respectively in case of relapse except for one neo adjuvant case. The majority of patients were older than 50. The tumors were primarily HGS ovarian tumors (88%), with 10% CC, and 2% endometrioid sub-types. Most of the tumors (88%) were stage III and IV. Majority of patients had a recurrent disease with a PFI of longer than 6 months (80%), while 20% of patients progressed or had a recurrence within 6 months.

Characteristics (patients, n=41) paired sections (82)	Range	Patient studied population	
		n/Total	%
Age (years)	≤ 50	13/41	32
	50 < Age ≤ 60	18/41	44
	> 60	10/41	24
FIGO stage	I	4/41	10
	II	1/41	2
	III	32/41	78
	IV	4/41	10
Progression-free interval (PFI)	PFI > 12 m	25/41	60
	6 m < PFI ≤ 12 m	8/41	20
	6 m ≤ PFI	8/41	20
Histologic sub-type	High grade serous	36/41	88
	Clear cell	4/41	10
	Endometrioid	1/41	2

Table S4. Antibodies used in the present studies. *Western Blotting (WB), Immunofluorescence (IF), Monoclonal antibody (mAb), Polyclonal antibody (pAb), H (Human), R (Rabbit), M (Mouse), Mk (Monkey), Cell Signaling Technology (Danvers, MA, USA), Santa Cruz Biotechnology (Santa Cruz, CA, USA), Abcam (Cambridge, MA, USA),, Thermo Fisher Scientific (Waltham, MA, USA).

A. Primary antibodies

Antigen	Species Cross- reac-tivity	Host Species	Dilution	Company	Catalogue No.	Application
P-p53(Ser15)	H, M, R, Mk	Rabbit pAb	1/2000 (WB) 1/200 (IF) 1/80 (PLA)	Cell Signaling	9284	WB, IF, ChIP
P-p53(Ser15)	H	Mouse mAb	1/80 (PLA)	Cell Signaling	9286	IF
p53(DO-1)	H	Mouse mAb	1/200 (WB)	Santa Cruz	sc-126	WB, IF
AIF	H, M, R	Mouse mAb	1/80 (PLA)	Santa Cruz	sc-55519	PLA
TOM20	H	Mouse mAb	1/200	Santa Cruz	sc-17764	IF
β-Actin	H, M, R, Mk	Mouse mAb	1/4,000	Ab cam	Ab8226	WB

B. Secondary antibodies

Host/Conjugate	Dilution	Company	Cat No.	Application
Goat Anti-Rabbit Alexa Fluor 555 conjugate	1/400	Thermo Fisher Scientific	A21428	IF
Goat Anti-Mouse Alexa Fluor 488 conjugate	1/400		A11001	IF
Goat Anti-Rabbit Alexa Fluor 647 conjugate	1/400		A27040	IF
Goat-Anti Rabbit Alexa Fluor 680 conjugate	1/10,000		A27042	WB (Li-cor)
Goat-Anti Mouse Alexa Fluor 800 conjugate	1/8000		A32730	WB (Li-cor)

References

1. Fraser M, Bai T, Tsang BK. Akt promotes cisplatin resistance in human ovarian cancer cells through inhibition of p53 phosphorylation and nuclear function. International journal of cancer. 2008; 122: 534-46.
2. Abedini MR, Wang PW, Huang YF, Cao M, Chou CY, Shieh DB, et al. Cell fate regulation by gelsolin in human gynecologic cancers. Proceedings of the National Academy of Sciences of the United States of America. 2014; 111: 14442-7.
3. Anglesio MS, Wiegand KC, Melnyk N, Chow C, Salamanca C, Prentice LM, et al. Type-specific cell line models for type-specific ovarian cancer research. PloS one. 2013; 8: e72162.
4. Domcke, S.; Sinha, R.; Levine, D.A.; Sander, C.; Schultz, N. Evaluating cell lines as tumour models by comparison of genomic profiles. *Nature communications* **2013**, *4*, 2126, doi:10.1038/ncomms3126.
5. Leroy B, Girard L, Hollestelle A, Minna JD, Gazdar AF, Soussi T. Analysis of TP53 mutation status in human cancer cell lines: a reassessment. Human mutation. 2014; 35: 756-65.
