# Supplementary figures and images for: Nuclear HKII-P-p53 (Ser15) Interaction is a Prognostic Biomarker for Chemoresponsiveness and Glycolytic Regulation in Epithelial Ovarian Cancer
Source: Cancers (Basel). 2021 Jul 7;13(14):3399. doi: 10.3390/cancers13143399 (PMC8306240; doi:10.3390/cancers13143399)

**Figure 4**

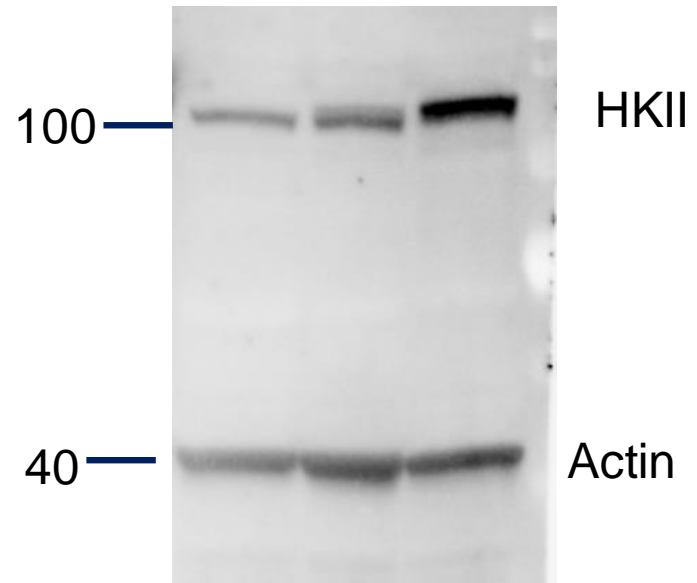

**Figure 5B**

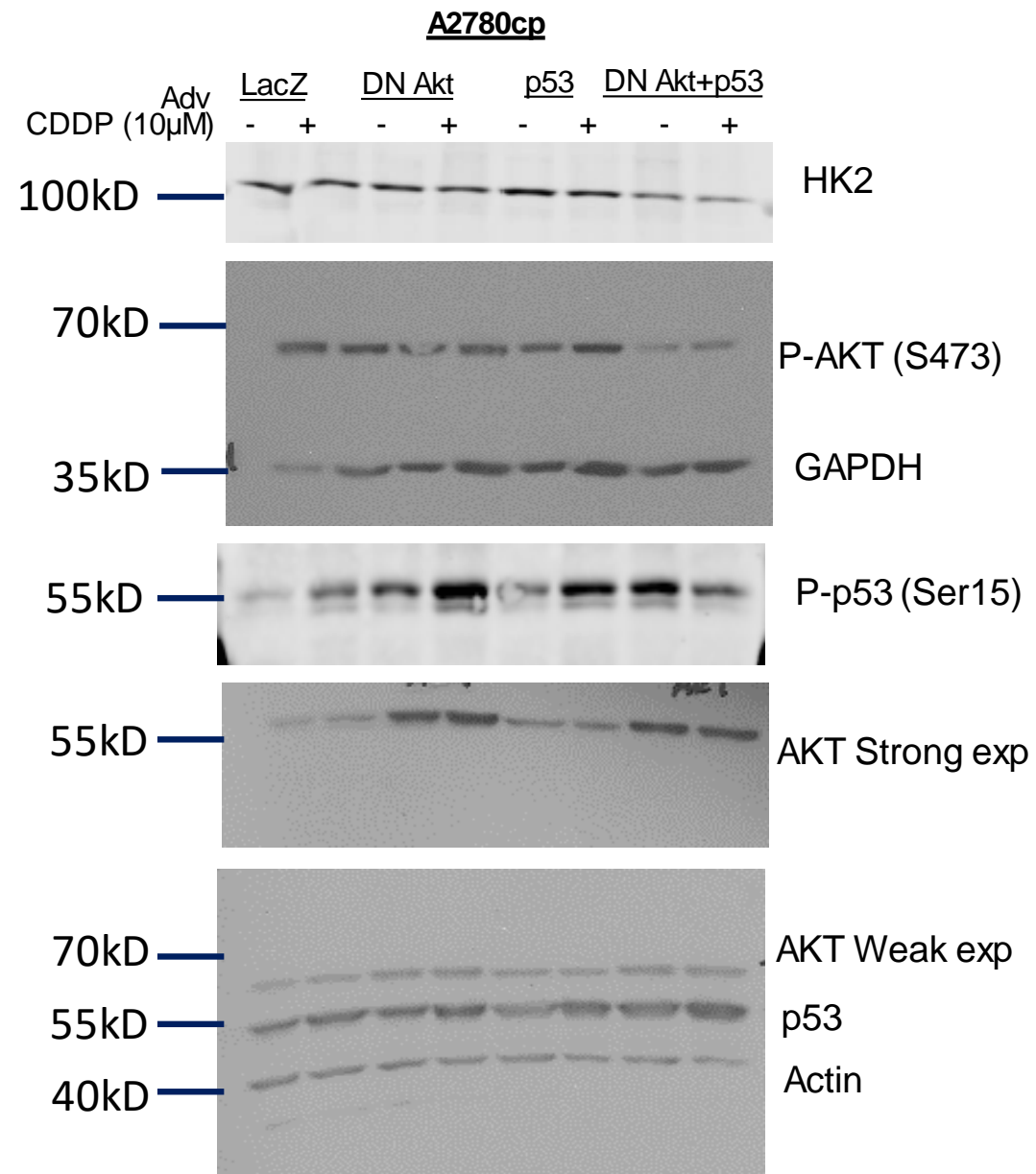

Figure 6E

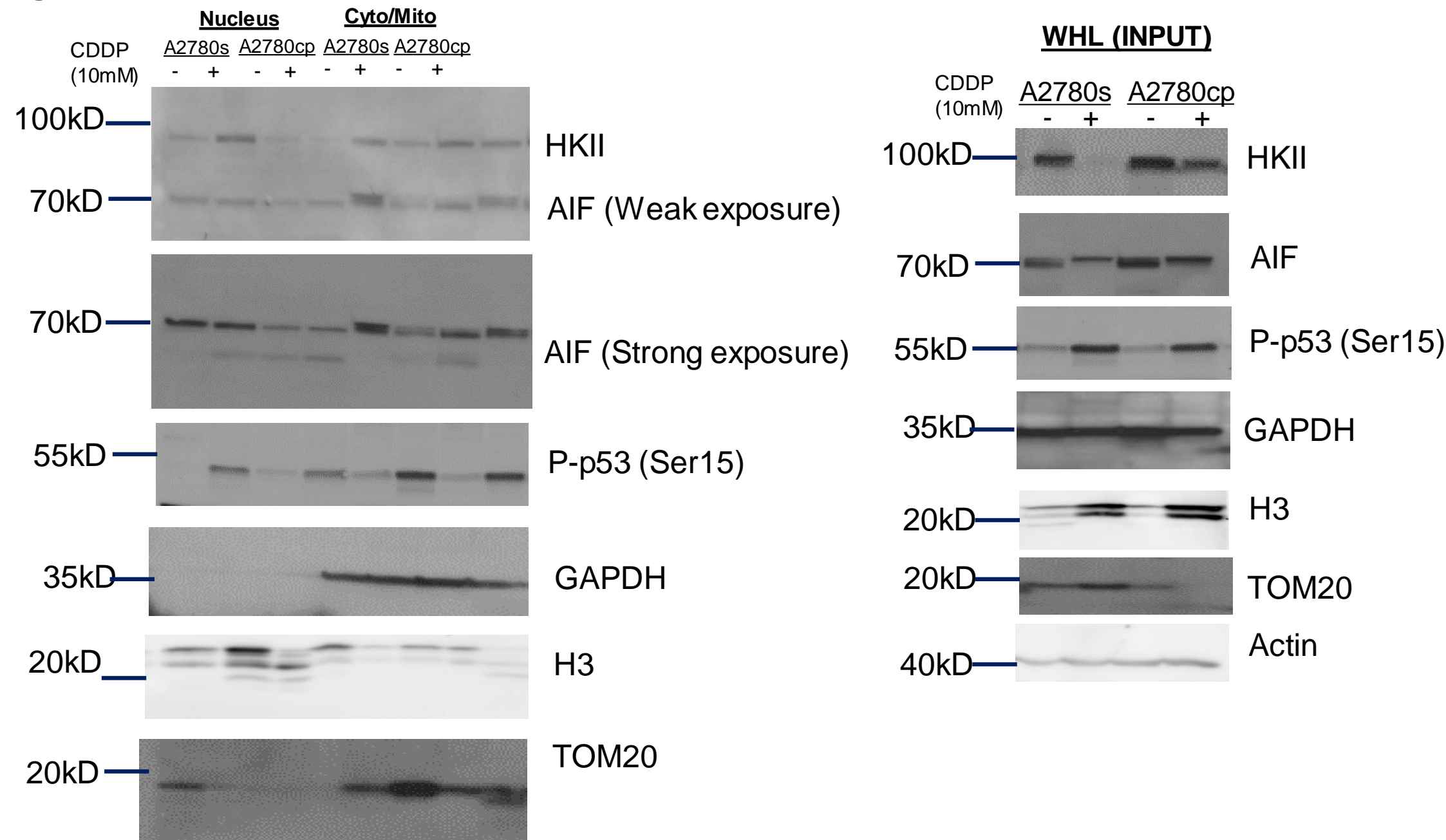

**Figure 6E**

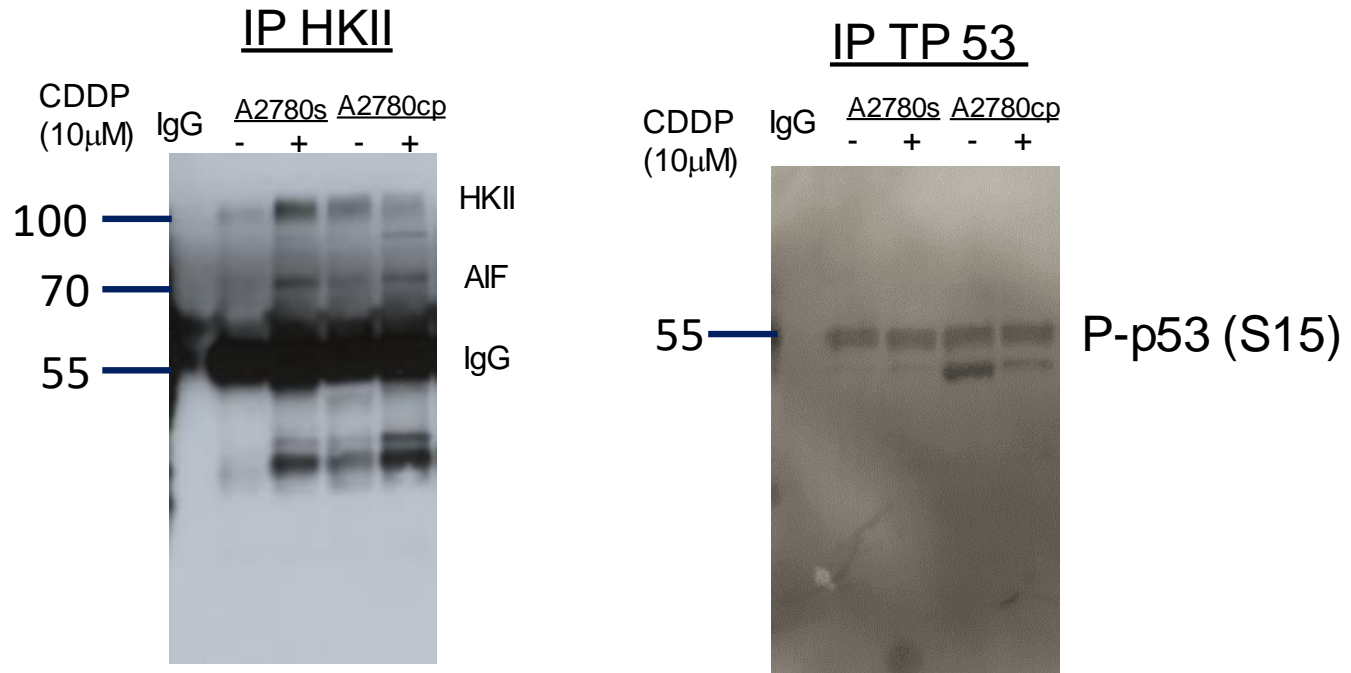

\*After incubating with true blot 2<sup>nd</sup> Ab

SI Figure S3(E)

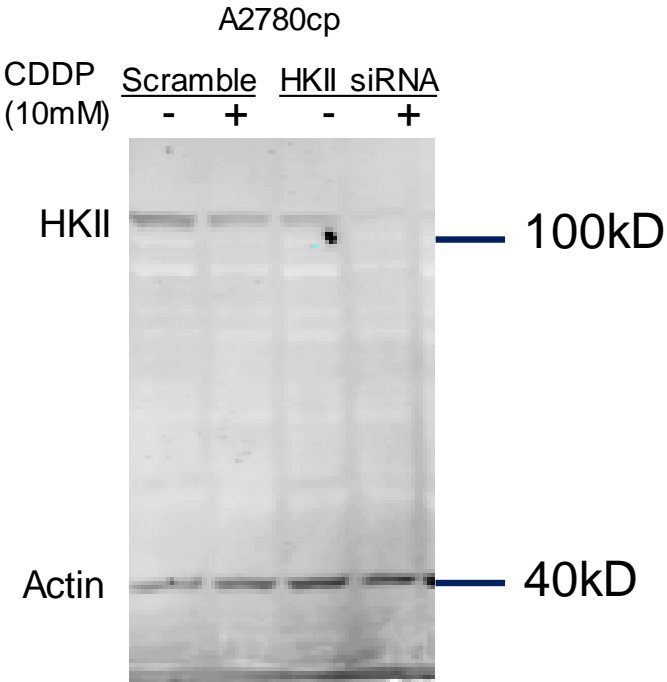

Supplement: Supplementary file 1 [file cancers-13-03399-s001.zip › cancers-1276270-supplementary/suppl/cancers-1276270-original-images.pdf]
